# {2,6-Bis[(4-bromo­phen­yl)imino­meth­yl]pyridine-κ^3^ *N*,*N*′,*N*′′}trichlorido­chromium(III)

**DOI:** 10.1107/S1600536810034689

**Published:** 2010-09-08

**Authors:** Xiao-Ping Li, Yong-Yong Liu, Jian-She Zhao

**Affiliations:** a Department of Chemistry, Shaanxi Key Laboratory for Physico-Inorganic Chemistry, Northwest University, Xi’an 710069, People’s Republic of China

## Abstract

In the title compound, [CrCl_3_(C_19_H_13_Br_2_N_3_)], the Cr^3+ ^ion is coordinated by the tridentate 2,6-bis­[(4-bromo­phen­yl)imino­meth­yl]pyridine Schiff base ligand in a *fac*-octa­hedral geometry. The dihedral angles between the pyridine and benzene rings are 23.9 (6) and 70.7 (1)°.

## Related literature

For background to Schiff bases as chelating ligands, see: Yin *et al.* (2010 ▶); Yang *et al.* (2010 ▶); Barboiu *et al.* (2009 ▶); Rohini *et al.* (2009 ▶); Legrand *et al.* (2009 ▶). For similar zinc complexes, see: Ceniceros-Gomez *et al.* (2000 ▶); Sugiyama *et al.* (2002 ▶); Sun *et al.* (2009 ▶); Gong *et al.* (2009 ▶); Xiao *et al.* (2010 ▶).
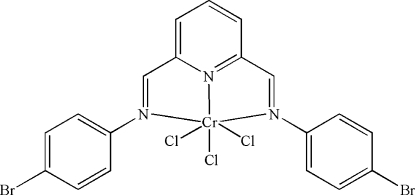


## Experimental

### 

#### Crystal data

[CrCl_3_(C_19_H_13_Br_2_N_3_)]
*M* *_r_* = 601.49
Monoclinic, 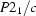

*a* = 13.722 (3) Å
*b* = 10.111 (2) Å
*c* = 18.905 (3) Å
β = 124.702 (12)°
*V* = 2156.4 (7) Å^3^
*Z* = 4
Mo *K*α radiation
μ = 4.62 mm^−1^
*T* = 296 K
0.20 × 0.13 × 0.09 mm

#### Data collection

Bruker APEXII CCD diffractometer
Absorption correction: multi-scan (*SADABS*; Sheldrick, 1996 ▶) *T*_min_ = 0.457, *T*_max_ = 0.689
11189 measured reflections
4177 independent reflections
2279 reflections with *I* > 2σ(*I*)
*R*_int_ = 0.053

#### Refinement

*R*[*F* ^2^ > 2σ(*F* ^2^)] = 0.047
*wR*(*F* ^2^) = 0.153
*S* = 0.97
4177 reflections
254 parameters
H-atom parameters constrained
Δρ_max_ = 0.61 e Å^−3^
Δρ_min_ = −0.41 e Å^−3^

### 

Data collection: *APEX2* (Bruker, 2004 ▶); cell refinement: *SAINT* (Bruker, 2004 ▶); data reduction: *SAINT*; program(s) used to solve structure: *SHELXS97* (Sheldrick, 2008 ▶); program(s) used to refine structure: *SHELXL97* (Sheldrick, 2008 ▶); molecular graphics: *SHELXTL* (Sheldrick, 2008 ▶); software used to prepare material for publication: *SHELXTL*.

## Supplementary Material

Crystal structure: contains datablocks I, global. DOI: 10.1107/S1600536810034689/ng5023sup1.cif

Structure factors: contains datablocks I. DOI: 10.1107/S1600536810034689/ng5023Isup2.hkl

Additional supplementary materials:  crystallographic information; 3D view; checkCIF report

## Figures and Tables

**Fig. 1.**
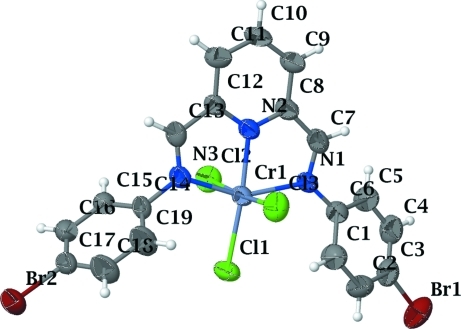
The molecular structure of the title complex, showing 50% probability displacement ellipsoids.

## supplementary crystallographic information

### Comment

Schiff bases have often been used as chelating ligands in coordination chemistry (Yin *et al.* (2010); Yang *et al.* (2010); Rohini *et al.* (2009): Legrand *et al.* (2009)). We report here the crystal structure of the title new chromium complex with the chelating Schiff base ligand 2, 6-bis [1-(4-bromophenylimino)] pyridine The Cr atom in the complex is six-coordinated by one pyridine N and two imine N atoms of the Schiff base ligand, and by two bromide atoms, forming tetrahedral geometry (Fig.1). The dihedral angle between the pyridine and the benzene rings is 23.9 (6) ° and 70.7 (1) °. The bond lengths (Table 1) related to the Cr atom are comparable to those observed in similar chromium complexes (Sugiyama *et al.* (2002); Sun *et al.* (2009); Gong *et al.* (2009); Xiao *et al.* (2010)).

### Experimental

2,6-bis[1-(4-bromophenylimino)]pyridine(0.0226 g, 0.05 mmol), and CrCl_3_?6H_2_O (0.0139 g, 0.05 mmol) were mixed and stirred in ethanol(10 ml) for 2 min and then heated in a stainless steel reactor with Teflon liner at 353 K for 72 h. After cooling at 5 K per hour, green crystals were obtained.

### Refinement

H atoms were positioned geometrically(C—H = 0.93 Å) and refined using a riding model, with *U*_iso_ (H) = 1.2Ueq(C).

### Crystal data

**Table:**

[CrCl_3_(C_19_H_13_Br_2_N_3_)]	*F*(000) = 1172
*M**_r_* = 601.49	*D*_x_ = 1.853 Mg m^−^^3^
Monoclinic, *P*2_1_/*c*	Mo *K*α radiation, λ = 0.71073 Å
Hall symbol: -P 2ybc	Cell parameters from 1555 reflections
*a* = 13.722 (3) Å	θ = 2.4–19.9°
*b* = 10.111 (2) Å	µ = 4.62 mm^−^^1^
*c* = 18.905 (3) Å	*T* = 296 K
β = 124.702 (12)°	Block, green
*V* = 2156.4 (7) Å^3^	0.20 × 0.13 × 0.09 mm
*Z* = 4	

### Data collection

**Table:**

Bruker APEXII CCD diffractometer	4177 independent reflections
Radiation source: fine-focus sealed tube	2279 reflections with *I* > 2σ(*I*)
graphite	*R*_int_ = 0.053
φ and ω scans	θ_max_ = 25.9°, θ_min_ = 2.2°
Absorption correction: multi-scan (*SADABS*; Sheldrick, 1996)	*h* = −16→13
*T*_min_ = 0.457, *T*_max_ = 0.689	*k* = −12→12
11189 measured reflections	*l* = −23→23

### Refinement

**Table:**

Refinement on *F*^2^	Secondary atom site location: difference Fourier map
Least-squares matrix: full	Hydrogen site location: inferred from neighbouring sites
*R*[*F*^2^ > 2σ(*F*^2^)] = 0.047	H-atom parameters constrained
*wR*(*F*^2^) = 0.153	*w* = 1/[σ^2^(*F*_o_^2^) + (0.0769*P*)^2^] where *P* = (*F*_o_^2^ + 2*F*_c_^2^)/3
*S* = 0.97	(Δ/σ)_max_ = 0.001
4177 reflections	Δρ_max_ = 0.61 e Å^−^^3^
254 parameters	Δρ_min_ = −0.41 e Å^−^^3^
0 restraints	Extinction correction: *SHELXL97* (Sheldrick, 2008), Fc^*^=kFc[1+0.001xFc^2^λ^3^/sin(2θ)]^-1/4^
Primary atom site location: structure-invariant direct methods	Extinction coefficient: 0.0012 (4)

### Special details

**Table:**

Geometry. All e.s.d.'s (except the e.s.d. in the dihedral angle between two l.s. planes) are estimated using the full covariance matrix. The cell e.s.d.'s are taken into account individually in the estimation of e.s.d.'s in distances, angles and torsion angles; correlations between e.s.d.'s in cell parameters are only used when they are defined by crystal symmetry. An approximate (isotropic) treatment of cell e.s.d.'s is used for estimating e.s.d.'s involving l.s. planes.
Refinement. Refinement of *F*^2^ against ALL reflections. The weighted *R*-factor *wR* and goodness of fit *S* are based on *F*^2^, conventional *R*-factors *R* are based on *F*, with *F* set to zero for negative *F*^2^. The threshold expression of *F*^2^ > σ(*F*^2^) is used only for calculating *R*-factors(gt) *etc*. and is not relevant to the choice of reflections for refinement. *R*-factors based on *F*^2^ are statistically about twice as large as those based on *F*, and *R*- factors based on ALL data will be even larger.

### Fractional atomic coordinates and isotropic or equivalent isotropic displacement parameters (Å^2^)

**Table:**

	*x*	*y*	*z*	*U*_iso_*/*U*_eq_	
Cr1	0.28797 (8)	0.86124 (8)	0.64659 (6)	0.0434 (3)	
Br1	0.69677 (8)	0.65971 (9)	1.11306 (5)	0.0892 (3)	
Br2	0.00271 (10)	0.39656 (7)	0.26880 (5)	0.1018 (4)	
Cl1	0.31007 (19)	0.63611 (14)	0.66454 (12)	0.0757 (6)	
Cl2	0.44502 (15)	0.87626 (17)	0.63412 (11)	0.0630 (5)	
Cl3	0.12377 (15)	0.86383 (17)	0.65121 (11)	0.0656 (5)	
N1	0.3903 (4)	0.9528 (4)	0.7728 (3)	0.0483 (12)	
N2	0.2615 (4)	1.0541 (4)	0.6191 (3)	0.0449 (11)	
N3	0.1766 (4)	0.8645 (4)	0.5113 (3)	0.0463 (12)	
C1	0.4323 (7)	0.7618 (7)	0.8620 (4)	0.0691 (19)	
H1	0.3679	0.7197	0.8145	0.083*	
C2	0.5019 (8)	0.6955 (7)	0.9398 (5)	0.077 (2)	
H2	0.4839	0.6084	0.9440	0.092*	
C3	0.5961 (6)	0.7561 (7)	1.0101 (4)	0.0632 (18)	
C4	0.6215 (6)	0.8848 (7)	1.0040 (4)	0.071 (2)	
H4	0.6850	0.9269	1.0522	0.085*	
C5	0.5537 (6)	0.9535 (7)	0.9267 (4)	0.0658 (18)	
H5	0.5721	1.0407	0.9232	0.079*	
C6	0.4597 (5)	0.8921 (6)	0.8557 (4)	0.0480 (14)	
C7	0.3845 (6)	1.0794 (6)	0.7687 (4)	0.0560 (16)	
H7	0.4241	1.1302	0.8185	0.067*	
C8	0.3151 (5)	1.1420 (5)	0.6847 (4)	0.0501 (15)	
C9	0.2975 (6)	1.2779 (6)	0.6666 (4)	0.0571 (17)	
H9	0.3329	1.3398	0.7109	0.069*	
C10	0.2260 (6)	1.3180 (6)	0.5810 (4)	0.0591 (17)	
H10	0.2134	1.4078	0.5680	0.071*	
C11	0.1731 (5)	1.2261 (6)	0.5144 (4)	0.0531 (16)	
H11	0.1258	1.2530	0.4573	0.064*	
C12	0.1932 (5)	1.0924 (5)	0.5360 (3)	0.0424 (13)	
C13	0.1483 (5)	0.9800 (6)	0.4780 (4)	0.0480 (14)	
H13	0.1007	0.9917	0.4187	0.058*	
C14	0.1391 (5)	0.7505 (5)	0.4560 (3)	0.0436 (14)	
C15	0.1856 (6)	0.7279 (6)	0.4095 (4)	0.0498 (15)	
H15	0.2436	0.7837	0.4152	0.060*	
C16	0.1458 (7)	0.6218 (6)	0.3541 (4)	0.0623 (19)	
H16	0.1769	0.6053	0.3222	0.075*	
C17	0.0601 (7)	0.5402 (6)	0.3461 (4)	0.0626 (19)	
C18	0.0162 (7)	0.5603 (7)	0.3942 (5)	0.077 (2)	
H18	−0.0405	0.5031	0.3891	0.092*	
C19	0.0562 (6)	0.6658 (6)	0.4505 (5)	0.070 (2)	
H19	0.0277	0.6796	0.4842	0.084*	

### Atomic displacement parameters (Å^2^)

**Table:**

	*U*^11^	*U*^22^	*U*^33^	*U*^12^	*U*^13^	*U*^23^
Cr1	0.0463 (6)	0.0347 (5)	0.0413 (5)	−0.0011 (4)	0.0203 (5)	−0.0050 (4)
Br1	0.0738 (6)	0.1233 (8)	0.0643 (5)	0.0183 (5)	0.0356 (5)	0.0310 (5)
Br2	0.1720 (11)	0.0517 (5)	0.0608 (5)	−0.0254 (5)	0.0540 (6)	−0.0206 (4)
Cl1	0.1130 (17)	0.0359 (9)	0.0707 (12)	0.0020 (9)	0.0479 (12)	−0.0021 (8)
Cl2	0.0507 (10)	0.0704 (11)	0.0663 (11)	0.0064 (9)	0.0324 (9)	0.0027 (8)
Cl3	0.0572 (11)	0.0812 (12)	0.0614 (10)	−0.0099 (9)	0.0355 (9)	−0.0088 (9)
N1	0.055 (3)	0.037 (3)	0.044 (3)	−0.002 (2)	0.023 (3)	−0.006 (2)
N2	0.047 (3)	0.036 (3)	0.042 (3)	−0.001 (2)	0.020 (2)	−0.005 (2)
N3	0.047 (3)	0.041 (3)	0.041 (3)	−0.004 (2)	0.018 (3)	−0.007 (2)
C1	0.070 (5)	0.064 (4)	0.055 (4)	−0.006 (4)	0.024 (4)	−0.001 (4)
C2	0.098 (6)	0.056 (4)	0.067 (5)	−0.003 (4)	0.041 (5)	0.010 (4)
C3	0.058 (5)	0.077 (5)	0.047 (4)	0.013 (4)	0.026 (4)	0.004 (4)
C4	0.061 (5)	0.086 (5)	0.046 (4)	−0.012 (4)	0.019 (4)	−0.010 (4)
C5	0.059 (4)	0.065 (4)	0.049 (4)	−0.011 (4)	0.017 (4)	0.000 (3)
C6	0.045 (4)	0.046 (3)	0.047 (4)	0.002 (3)	0.022 (3)	−0.002 (3)
C7	0.058 (4)	0.053 (4)	0.044 (4)	−0.002 (3)	0.021 (3)	−0.011 (3)
C8	0.048 (4)	0.039 (3)	0.049 (4)	−0.004 (3)	0.020 (3)	−0.006 (3)
C9	0.060 (4)	0.038 (3)	0.054 (4)	0.002 (3)	0.022 (4)	−0.002 (3)
C10	0.066 (5)	0.030 (3)	0.071 (5)	−0.003 (3)	0.033 (4)	−0.002 (3)
C11	0.049 (4)	0.042 (3)	0.058 (4)	0.002 (3)	0.024 (3)	0.006 (3)
C12	0.043 (4)	0.036 (3)	0.042 (3)	−0.002 (3)	0.020 (3)	−0.005 (3)
C13	0.042 (4)	0.054 (4)	0.040 (3)	−0.006 (3)	0.019 (3)	−0.010 (3)
C14	0.047 (4)	0.042 (3)	0.033 (3)	−0.005 (3)	0.017 (3)	−0.009 (3)
C15	0.063 (4)	0.042 (3)	0.046 (4)	−0.004 (3)	0.033 (3)	−0.002 (3)
C16	0.097 (6)	0.044 (4)	0.057 (4)	0.003 (4)	0.050 (4)	0.001 (3)
C17	0.094 (6)	0.040 (3)	0.036 (3)	0.002 (4)	0.026 (4)	−0.002 (3)
C18	0.079 (5)	0.060 (5)	0.077 (5)	−0.024 (4)	0.036 (5)	−0.023 (4)
C19	0.068 (5)	0.066 (5)	0.084 (5)	−0.016 (4)	0.048 (5)	−0.021 (4)

### Geometric parameters (Å, °)

**Table:**

Cr1—N2	1.997 (4)	C5—H5	0.9300
Cr1—N3	2.105 (5)	C7—C8	1.451 (8)
Cr1—N1	2.170 (5)	C7—H7	0.9300
Cr1—Cl1	2.2959 (17)	C8—C9	1.403 (8)
Cr1—Cl2	2.3025 (19)	C9—C10	1.394 (8)
Cr1—Cl3	2.3042 (19)	C9—H9	0.9300
Br1—C3	1.893 (6)	C10—C11	1.391 (8)
Br2—C17	1.886 (6)	C10—H10	0.9300
N1—C7	1.283 (7)	C11—C12	1.393 (8)
N1—C6	1.428 (7)	C11—H11	0.9300
N2—C12	1.349 (7)	C12—C13	1.450 (7)
N2—C8	1.353 (7)	C13—H13	0.9300
N3—C13	1.277 (7)	C14—C15	1.369 (7)
N3—C14	1.439 (6)	C14—C19	1.379 (8)
C1—C2	1.387 (9)	C15—C16	1.377 (8)
C1—C6	1.394 (8)	C15—H15	0.9300
C1—H1	0.9300	C16—C17	1.372 (9)
C2—C3	1.363 (9)	C16—H16	0.9300
C2—H2	0.9300	C17—C18	1.361 (9)
C3—C4	1.369 (9)	C18—C19	1.381 (8)
C4—C5	1.392 (9)	C18—H18	0.9300
C4—H4	0.9300	C19—H19	0.9300
C5—C6	1.370 (8)		
			
N2—Cr1—N3	76.68 (18)	C1—C6—N1	117.1 (6)
N2—Cr1—N1	77.16 (18)	N1—C7—C8	118.8 (5)
N3—Cr1—N1	153.84 (18)	N1—C7—H7	120.6
N2—Cr1—Cl1	174.56 (14)	C8—C7—H7	120.6
N3—Cr1—Cl1	97.97 (13)	N2—C8—C9	119.5 (5)
N1—Cr1—Cl1	108.18 (14)	N2—C8—C7	113.1 (5)
N2—Cr1—Cl2	87.19 (14)	C9—C8—C7	127.4 (6)
N3—Cr1—Cl2	87.05 (14)	C10—C9—C8	118.5 (6)
N1—Cr1—Cl2	91.55 (14)	C10—C9—H9	120.7
Cl1—Cr1—Cl2	91.60 (7)	C8—C9—H9	120.7
N2—Cr1—Cl3	87.83 (14)	C11—C10—C9	121.1 (6)
N3—Cr1—Cl3	89.83 (14)	C11—C10—H10	119.5
N1—Cr1—Cl3	89.31 (14)	C9—C10—H10	119.5
Cl1—Cr1—Cl3	93.18 (7)	C10—C11—C12	118.0 (6)
Cl2—Cr1—Cl3	174.62 (7)	C10—C11—H11	121.0
C7—N1—C6	118.4 (5)	C12—C11—H11	121.0
C7—N1—Cr1	112.3 (4)	N2—C12—C11	120.7 (5)
C6—N1—Cr1	129.3 (4)	N2—C12—C13	111.7 (5)
C12—N2—C8	122.2 (5)	C11—C12—C13	127.6 (5)
C12—N2—Cr1	119.1 (4)	N3—C13—C12	117.7 (5)
C8—N2—Cr1	118.6 (4)	N3—C13—H13	121.2
C13—N3—C14	119.5 (5)	C12—C13—H13	121.2
C13—N3—Cr1	114.8 (4)	C15—C14—C19	120.9 (5)
C14—N3—Cr1	125.6 (4)	C15—C14—N3	119.7 (5)
C2—C1—C6	119.4 (7)	C19—C14—N3	119.4 (5)
C2—C1—H1	120.3	C14—C15—C16	119.4 (6)
C6—C1—H1	120.3	C14—C15—H15	120.3
C3—C2—C1	121.0 (7)	C16—C15—H15	120.3
C3—C2—H2	119.5	C17—C16—C15	119.7 (6)
C1—C2—H2	119.5	C17—C16—H16	120.1
C2—C3—C4	119.4 (6)	C15—C16—H16	120.1
C2—C3—Br1	120.2 (6)	C18—C17—C16	121.0 (6)
C4—C3—Br1	120.3 (6)	C18—C17—Br2	118.9 (5)
C3—C4—C5	120.9 (6)	C16—C17—Br2	120.1 (5)
C3—C4—H4	119.6	C17—C18—C19	119.8 (7)
C5—C4—H4	119.6	C17—C18—H18	120.1
C6—C5—C4	119.7 (6)	C19—C18—H18	120.1
C6—C5—H5	120.1	C14—C19—C18	119.2 (6)
C4—C5—H5	120.1	C14—C19—H19	120.4
C5—C6—C1	119.6 (6)	C18—C19—H19	120.4
C5—C6—N1	123.2 (5)		
			
N2—Cr1—N1—C7	−0.1 (4)	C7—N1—C6—C5	−26.0 (9)
N3—Cr1—N1—C7	0.2 (7)	Cr1—N1—C6—C5	154.0 (5)
Cl1—Cr1—N1—C7	178.9 (4)	C7—N1—C6—C1	157.2 (6)
Cl2—Cr1—N1—C7	86.7 (4)	Cr1—N1—C6—C1	−22.8 (8)
Cl3—Cr1—N1—C7	−88.0 (4)	C6—N1—C7—C8	179.5 (5)
N2—Cr1—N1—C6	179.9 (5)	Cr1—N1—C7—C8	−0.6 (7)
N3—Cr1—N1—C6	−179.8 (4)	C12—N2—C8—C9	1.4 (9)
Cl1—Cr1—N1—C6	−1.1 (5)	Cr1—N2—C8—C9	−180.0 (4)
Cl2—Cr1—N1—C6	−93.3 (5)	C12—N2—C8—C7	−179.9 (5)
Cl3—Cr1—N1—C6	92.0 (5)	Cr1—N2—C8—C7	−1.3 (7)
N3—Cr1—N2—C12	−0.5 (4)	N1—C7—C8—N2	1.2 (8)
N1—Cr1—N2—C12	179.4 (4)	N1—C7—C8—C9	179.8 (6)
Cl1—Cr1—N2—C12	9.9 (19)	N2—C8—C9—C10	−0.7 (9)
Cl2—Cr1—N2—C12	87.2 (4)	C7—C8—C9—C10	−179.2 (6)
Cl3—Cr1—N2—C12	−90.8 (4)	C8—C9—C10—C11	−0.2 (10)
N3—Cr1—N2—C8	−179.1 (5)	C9—C10—C11—C12	0.4 (9)
N1—Cr1—N2—C8	0.8 (4)	C8—N2—C12—C11	−1.2 (8)
Cl1—Cr1—N2—C8	−168.7 (14)	Cr1—N2—C12—C11	−179.8 (4)
Cl2—Cr1—N2—C8	−91.5 (4)	C8—N2—C12—C13	178.6 (5)
Cl3—Cr1—N2—C8	90.5 (4)	Cr1—N2—C12—C13	0.0 (6)
N2—Cr1—N3—C13	0.9 (4)	C10—C11—C12—N2	0.2 (9)
N1—Cr1—N3—C13	0.6 (7)	C10—C11—C12—C13	−179.5 (6)
Cl1—Cr1—N3—C13	−178.1 (4)	C14—N3—C13—C12	−176.8 (5)
Cl2—Cr1—N3—C13	−86.9 (4)	Cr1—N3—C13—C12	−1.2 (7)
Cl3—Cr1—N3—C13	88.7 (4)	N2—C12—C13—N3	0.8 (7)
N2—Cr1—N3—C14	176.2 (5)	C11—C12—C13—N3	−179.4 (6)
N1—Cr1—N3—C14	175.9 (4)	C13—N3—C14—C15	67.8 (7)
Cl1—Cr1—N3—C14	−2.8 (4)	Cr1—N3—C14—C15	−107.2 (5)
Cl2—Cr1—N3—C14	88.4 (4)	C13—N3—C14—C19	−111.8 (7)
Cl3—Cr1—N3—C14	−96.0 (4)	Cr1—N3—C14—C19	73.1 (7)
C6—C1—C2—C3	0.3 (11)	C19—C14—C15—C16	2.3 (9)
C1—C2—C3—C4	0.8 (11)	N3—C14—C15—C16	−177.3 (5)
C1—C2—C3—Br1	−175.3 (5)	C14—C15—C16—C17	0.1 (9)
C2—C3—C4—C5	−1.1 (11)	C15—C16—C17—C18	−2.0 (10)
Br1—C3—C4—C5	174.9 (5)	C15—C16—C17—Br2	178.8 (5)
C3—C4—C5—C6	0.4 (10)	C16—C17—C18—C19	1.5 (11)
C4—C5—C6—C1	0.7 (10)	Br2—C17—C18—C19	−179.3 (5)
C4—C5—C6—N1	−176.0 (6)	C15—C14—C19—C18	−2.9 (10)
C2—C1—C6—C5	−1.0 (10)	N3—C14—C19—C18	176.8 (6)
C2—C1—C6—N1	175.9 (6)	C17—C18—C19—C14	0.9 (11)
